# Genetics of extra‐early‐maturing yellow and orange quality protein maize inbreds and derived hybrids under low soil nitrogen and *Striga* infestation

**DOI:** 10.1002/csc2.20384

**Published:** 2020-12-22

**Authors:** P. Abu, B. Badu‐Apraku, B. E. Ifie, P. Tongoona, P. F. Ribeiro, E. Obeng‐Bio, S. K. Offei

**Affiliations:** ^1^ West Africa Centre for Crop Improvement Univ. of Ghana PMB 30 Legon Ghana; ^2^ International Institute of Tropical Agriculture (IITA) PMB 5320, Oyo Rd Ibadan Nigeria; ^3^ CSIR–Crops Research Institute PO Box 3785, Fumesua Kumasi Ghana

## Abstract

The development and commercialization of extra‐early quality protein maize (QPM)–provitamin A (PVA) hybrids that are tolerant of low soil N (LN) and *Striga* resistant are essential for addressing the food insecurity and undernourishment challenges currently faced by sub‐Saharan Africa (SSA). This study was designed (a) to determine the genetic effects regulating grain yield (GY) and important secondary traits of extra‐early yellow and orange QPM–PVA inbred lines under LN, *Striga‐*infested, and high‐N (HN) conditions, (b) to investigate whether maternal genes influenced the inheritance of GY and other secondary traits, (c) to assess the GY and stability of the hybrids across the three management conditions, and (d) to examine the relationship between single nucleotide polymorphism (SNP) marker‐based genetic distances and GY. Twenty‐four inbred lines were used to produce ninety‐six single cross hybrids using the North Carolina Design II. The performance of the hybrids plus four checks was assessed across LN, *Striga*‐infested, and HN management conditions in Ghana and Nigeria in 2018. Additive genetic variances were preponderant over nonadditive genetic variances for GY and most secondary traits in each and across environments. TZEEQI 358 exhibited significant and positive male and female GCA effects for GY under LN, *Striga* infestation, HN, and across management conditions indicating that favorable alleles for GY could be donated by TZEEQI 358. Maternal effects regulated the inheritance of plant height under the *Striga‐*infested conditions. Genetic distances were associated with GY under LN, *Striga* infestation, and HN conditions. TZEEIORQ 58 **×** TZEEQI 397 demonstrated high GY and stability of performance; therefore, it should be further tested under multiple environments for commercialization.

**Abbreviations**
ASI: anthesis–silking interval
BG: between group hybrids
CM: conventional maize
DA: days to 50% anthesis
DAP: days after planting
DS: days to silking
EA: ear aspect
EHT: ear height
EPP: ears per plant
GCA: general combining ability
GGE: genotype and genotype × environment
HC: husk cover
HN: high soil nitrogen
IITA: International Institute of Tropical Agriculture
LN: low soil nitrogen
MIP: maize improvement program
NCD II: North Carolina Design II
PHT: plant height
QPM: quality protein maize
SCA: specific combining ability
SeqSNP: targeted genotyping by sequencing
SNP: single nucleotide polymorphism
SSA: sub‐Saharan Africa
STYG: stay‐green characteristic
WACCI: West Africa Centre for Crop Improvement Research field
WAP: weeks after planting
WG: within‐group hybrids.

## 1 INTRODUCTION

Maize (*Zea mays* L.) is the primary cereal crop in sub‐Saharan Africa (SSA) where it is a key source of food security and nutrition with ∼300 million smallholder farmers dependent on it for food and cash (Mathege, Smale, & Olwande, 2014). Despite the critical position maize occupies in SSA, the current yield of 2.01 t ha^−1^ lags the global estimate of 5.75 t ha^−1^ (FAOSTAT, 2017). Among the biotic and abiotic stress factors responsible for the low yields, *Striga hermonthica* (Delile) Benth. infestation and low soil N (LN) rank high, especially in the savanna zones where the co‐occurrence of the two stresses at different growth and developmental stages is a common phenomenon (Ertiro et al., 2017).

Low N has become a persistent problem in SSA due to the inherently poor soil fertility status accompanied by low fertilizer application levels due to high cost and lack of investment in tackling the abiotic stress (Morosini, Mendonça, Vidotti, & Fritsche‐neto, 2017). The low fertilizer application rate currently estimated at 15 kg ha^−1^ (IFA, 2019) has been attributed to low domestic production capacity and poor marketing and distribution infrastructure, which have resulted in high cost and inaccessibility. *Striga* has also become a serious biotic plague in the savanna zones of SSA due to the shift from the traditional cereal‐based farming system that typically integrated crop rotation and longer fallow periods, which maintained the *Striga* seed bank at plant tolerable levels (Oswald, 2005). The consequence is the exacerbated *Striga* seed bank levels threatening maize production and the livelihoods of millions of farmers (Oswald, 2005). Although several *Striga* control methods are available (Badu‐Apraku & Fakorede, 2017; Kim & Adetimirin, 1997), a combination of methods is usually required to effectively control the parasite (Yallou, Menkir, Adetimirin, & Kling, 2009). The cultivation of resistant varieties plays an integral role in *Striga* control as it reduces damage to the plant at tolerable levels, as well as decreases the *Striga* seed buildup in the soil (Menkir, Franco, Adpoju, & Bossey, 2012). Individually, LN and *Striga* can cause yield reduction of 10–50% (Amegbor, Badu‐Apraku, & Annor, 2017) and 30–90% (Ejeta, 2007), respectively. However, when both stresses occur simultaneously and susceptible genotypes are planted, the interaction effects could lead to 100% yield loss (Haussmann, Hess, Welz, & Geiger, 2000). Therefore, it is crucial to develop maize varieties with combined tolerance to LN and *Striga* to mitigate the effects of the two stresses whether they occur in isolation or together.

The conventional maize (CM) varieties commonly consumed in SSA are nutrient deficient because they lack optimum levels of proteins (Atlin et al., 2011) and provitamin A carotenoids (Muzhingi et al., 2011). The protein deficiency status in CM is due to low levels of two essential amino acids, lysine (1.5–2.5%), and tryptophan (0.025–0.05%) (Sofi, Wani, Rather, & Wani, 2009). Quality protein maize (QPM) is more nutritious because it contains ∼73% protein compared with the 46% in CM (Badu‐Apraku & Fakorede, 2017). Although some QPM hybrids with LN tolerance and *Striga* resistance are available to farmers in SSA, no commercialized extra‐early‐maturing (80–85 d) yellow and orange QPM hybrids with LN tolerance and *Striga* resistance are currently available. Therefore, a major objective of the International Institute for Tropical Agriculture‐Maize Improvement Program (IITA‐MIP) over the past decade has focused on developing maize inbred lines with multiple stress tolerances (LN, *Striga*, and drought). Three groups of extra‐early‐maturing QPM inbred lines (extra‐early white, extra‐early yellow, and extra‐early orange with favorable alleles for provitamin A) with contrasting tolerance and resistance levels to LN, *Striga* and drought stresses have recently been developed. However, little information is available on the combining ability, type of gene action, and role of maternal effects for grain yield and important traits of the QPM yellow and orange inbred lines under LN, *Striga* and high‐N (HN) conditions. Furthermore, the importance of marker‐based genetic distances in selecting parents for hybrid production has not been ascertained for this set of inbred lines. This information is a prerequisite for adopting appropriate breeding strategies to accelerate selection gains and determine parents for superior hybrid development (Makumbi, Betrán, Bänziger, & Ribaut, 2011). Even though several studies have reported on the gene action and the role of maternal genes in the inheritance of grain yield and important straits under LN and *Striga* infestation, the results have been inconsistent. Although Ertiro et al. (2017), Obeng‐Bio et al. (2019), and Oyekale, Badu‐Apraku, and Adetimirin (2020) reported the inheritance of grain yield under LN to be largely influenced by additive gene action, Badu‐Apraku et al. (2016), Mafouasson et al. (2017), and Annor, Badu‐Apraku, Nyadanu, Akromah, and Fakorede (2019) reported prominence of nonadditive genes. Under *Striga*, Badu‐Apraku et al. (2020) and Oyekale et al. (2020) established that additive gene effects played a greater role in the inheritance of grain yield, *Striga* damage syndrome rating, and *Striga* emergence, whereas Badu‐Apraku et al. (2016) documented superiority of nonadditive gene effects. Maternal effects were reported to influence the inheritance of grain yield and few traits under LN, *Striga*, and HN conditions by Oyekale et al. (2020), whereas Ifie, Badu‐Apraku, Gracen, and Danquah (2015) reported nonsignificant maternal effects under similar growing conditions. The inconsistencies in the literature indicate that the expression of genes for LN tolerance and *Striga* resistance or tolerance are germplasm dependent, making it prudent to determine the gene action for the newly developed QPM yellow and orange inbred lines. Although high marker‐based genetic distance does not guarantee maximum heterosis, Suwarno, Pixley, Palacios‐Rojas, Kaeppler, and Babu (2014) established that grouping of inbred lines based on higher single nucleotide polymorphism (SNP)‐based genetic distances could help achieve higher heterosis. It is therefore important to determine whether SNP‐based genetic distances influenced hybrid performance of these inbred lines, to inform parent selection for hybrid production. In addition, knowledge on genotype × environment interaction (GEI) for newly developed hybrids is critical as far as specific or broad adaptation is concerned (Yan & Kang, 2003). This study was therefore designed (a) to ascertain the gene action governing the inheritance of grain yield and other traits of extra‐early yellow and orange QPM inbred lines under LN, *Striga*, and HN management conditions, (b) to assess the role of maternal effects for grain yield and other traits, (c) to evaluate the yield and stability of selected hybrids across LN, *Striga*, and HN conditions, and (d) to examine the relationship between SNP‐based genetic distances and hybrid performance under the LN, *Striga*, and HN management conditions.

## 2 MATERIALS AND METHODS

### 2.1 Description of genetic materials

Sixty‐five inbred lines were selected from two IITA‐MIP inbred line panels derived from two different extra‐early populations: 99 TZEE‐Y Pop STR QPM (yellow endosperm QPM *Striga*‐resistant population) and 2009 TZEE‐OR2 STR QPM (orange endosperm QPM *Striga*‐resistant populations with favorable alleles for provitamin A). The tryptophan contents of the lines were determined in the Nutritional Quality and Plant Tissue Analysis Laboratory of the CIMMYT, Mexico. Although the protein quality of maize is determined by both the lysine and tryptophan contents, only tryptophan was assayed for this study due to high cost implications. Furthermore, a significant positive correlation exists between tryptophan and lysine (Nurit, Tiessen, Pixley, & Palacios‐Rojas, 2009). Based on the results of the laboratory analysis, 24 inbred lines with tryptophan content ranging from 0.07 to 0.09%, which met the optimum requirement level of 0.07% (Nurit et al., 2009), were selected for the present study (Supplemental Table S1). Sixteen of the selected lines were yellow endosperm QPM, while eight were orange endosperm QPM.

### 2.2 Generation of single‐cross hybrids

The 24 inbred lines were used to generate 96 single‐cross hybrids using the North Carolina Design II (NCD II) scheme (Comstock & Robinson, 1948). The yellow endosperm lines were divided into four sets while the orange endosperm lines were divided into two, for a total of six groups containing four lines each. The sets were arranged such that there were crosses between yellow sets only, orange sets only, and between yellow and orange sets. The arrangement also ensured that each of the six sets served as both male and female parents (Supplemental Table S1). To achieve this, the sets were used in crosses in two ways: (a) each set was used as males and crossed to another set that served as females, and (b) each set was subsequently used as females and crossed to another set that served as males. The hybrid generation was carried out at the research field of West Africa Centre for Crop Improvement (WACCI), University of Ghana‐Legon, during the major planting season of 2017.

**Core Ideas**

- Additive gene action conditions yield of extra‐early QPM inbreds under low N and *Striga*.
- Maternal effects regulate inheritance of plant height and *Striga* infestation.
- Genetic distances are significantly associated with grain yield under low N and *Striga*.
- TZEEQI 358 is a valuable resource for low N tolerance and *Striga* resistance in maize.
- TZEEIORQ 58 × TZEEQI 397 was high yielding and stable across low N, *Striga*, and high N.

### 2.3 Experimental field location, layout, and agronomic practices

The 96 hybrids plus four checks were planted in separate field trials under LN, *Striga* and HN management conditions in 12 environments in Ghana and Nigeria in 2018 (Supplemental Table S2) as follows. The LN experiments were carried out in three environments in Ghana during the major rainy season (April–August) and minor rainy season (September–November). The *Striga* experiments were conducted in four environments in the *Striga* endemic Savanna agroecologies of Ghana and Nigeria during the major growing season (June–November) of 2018. The HN trials were carried out in five environments in Ghana and Nigeria during the major and minor planting seasons of 2018. Two of the checks (TZEEQI 183 × TZEEQI 7 and TZEEQI 181 × TZEEQI 7) used in this study were extra‐early white endosperm QPM single‐cross hybrids identified as outstanding hybrids across multiple stress environments in a previous study (Annor & Badu‐Apraku, 2016), whereas the other two checks (TZEEIORQ 64 × TZEEIORQ 25 and TZEEIORQ 61 × TZEEIORQ 43) were extra‐early QPM‐provitamin A single‐cross hybrids characterized as multiple stress tolerant in the IITA‐MIP. The white and orange endosperm QPM hybrid checks were used because no extra‐early yellow QPM hybrid checks with multiple stresses tolerance were available at the time of the study.

The layout of the experiments followed a 10 × 10 α‐lattice designs with two replicates per experimental site. Plot size of 4‐m rows spaced at 0.4 m within rows and 0.75 m between rows was adopted for all experiments. Three seeds were sown per hill and thinning was subsequently done to maintain two plants per stand and achieve the maximum plant population density of 66,666 ha^−1^. Experimental sites designated for LN experiments were previously depleted of the N reserve by planting the field to maize at high population density without fertilizer application, and the aboveground biomass was then removed at full maturity. These sites had also been devoted to only LN trials over a few years. Soil samples were collected from all the LN experimental sites for analysis prior to planting to ensure that the N levels were low. Soil analysis followed the method described by Bremner and Mulvaney (1983). The soils at Fumesua, Legon, and Nyankpala contained 0.16, 0.05, and 0.05% N, respectively, and were considered low N levels since they were lower than 0.2% threshold according to Landon (1991) interpretation. The LN trials received the appropriate amount of N fertilizer (urea), depending on the initial N level, to achieve the target of 30 kg N ha^−1^ of available N while 90 kg N ha^−1^ was applied to the HN trials at 2 and 5 wk after planting (WAP). The LN and HN trials received 60 kg ha^−1^ of P and K respectively, applied as muriate of potash and triple super phosphate, respectively. Weeds in LN and HN trials were controlled manually by hand weeding. *Striga* fields were artificially infested following the procedure of the IITA‐MIP (Kim, 1991). At planting, each planting hole received 8.5 g of *Striga*–sand mixture containing about 5,000 *Striga* seeds with good germinability. Fertilization of the *Striga‐*inoculated maize plots was delayed up to about 25–30 d after planting to induce enough stress on the maize plants and stimulate production of strigolactones, a hormone that promotes the germination of *Striga* seeds and causes attachment of the emerging parasitic plants to the roots of maize plants (Kim, 1994). *Striga‐*infested fields were fertilized with 20–30 kg ha^−1^ of NPK. All weeds, excluding *Striga*, were controlled manually.

### 2.4 Genotyping of inbred lines using SNP markers

Leaf discs were taken from six to eight seedlings each of the 24 parents at 2 wk old and bulked together. The leaf samples were sent to LGC Company (http://www.lgcgroup.com/) for both DNA extraction and genotyping. Two thousand five hundred SNP markers were used to genotype the inbred lines via the targeted genotyping by sequencing (SeqSNP) platform available at LGC (https://www.biosearchtech.com/services/sequencing/targeted-genotyping-by-sequencing-seqsnp).

### 2.5 Data collected

The number of days to 50% anthesis (DA) and silking (DS) were determined by subtracting the planting date from the date on which half of the plants reached anthesis and silk appearance, respectively. Anthesis–silking interval (ASI) was determined by subtracting the DA from DS. Plant and ear heights were the distances between the base of the plant and the first tassel branch and the ear‐bearing upper node, respectively. Husk cover was rated using a scale of 1–5 where 1 denoted tightly layered husk stretching beyond the tip of the ear, and 5 denoted fully exposed ear tip. Ears per plant was estimated by dividing the total number of ears harvested in each plot by the number of plants harvested. Ear aspect was scored on a scale of 1–9 wherein 1 denoted disease‐free, large, uniform, and well‐ filled ears and 9 denoted ears with undesirable features (Badu‐Apraku et al., 2016). The following data were specific to low N trials only: (a) stay‐green characteristic, determined as the extent of leaf death scored at 70 d after planting (DAP) employing a scale of 1–9 where 1 denoted hybrids displaying 0–10% total dead leaf area and 9 denoted 81–100% dead leaf area; (b) plant aspect was scored on a scale of 1–9 with 1 denoting excellent overall phenotypic appearance and 9 denoting extremely poor overall phenotypic appearance (Badu‐Apraku et al., 2016). Chlorophyll content was recorded with a SPAD meter at 70 DAP, averaged for five random plants in each plot. Additional data recorded in *Striga*‐infested trials only were (a) number of emerged *Striga* plants as a count of *Striga* plants growing in each plot at 8 and 10 WAP; (b) *Striga* damage syndrome rating of host plant at 8 and 10 WAP using a scale of 1–9 with 1 denoting normal plant growth without any visible host plant damage and 9 denoting premature death or total failure of host plant without ear formation due to complete scorching of all leaves (Kim, 1991). Grain weight (kg ha^−1^) in LN plots was estimated using the weight and moisture content of the grains from shelled ears from each plot after adjusting the moisture content to 15%. Grain yield in HN and *Striga*‐infested plots was estimated on a plot basis assuming 80% shelling percentage with adjusted moisture content of 15%.

### 2.6 Data analyses

Data recorded for number of emerged *Striga* plants were log transformed [log(count + 1)] ahead of the ANOVA. Location and season combinations were considered as environments whereas the treatments (LN, *Striga*, and HN conditions) were considered as management conditions. Separate ANOVA was initially performed using the data for measured traits under LN, *Striga*, and HN management conditions. Subsequently, the data were subjected to the Bartlett's test of homogeneity of variances (Snedecor & Cochran, 1989) for the LN, *Striga*, and HN environments (location–year combinations), but the tests were not significant. Therefore, combined ANOVA was performed across the three management conditions using the data from all the 12 environments. The ANOVA used the general linear model procedure implemented as PROC GLM in SAS version 9.4 (SAS Institute, 2012). Adjustments were made for block effects on hybrid means according to the lattice design (Cochran & Cox, 1960). Environments, replications, and blocks were treated as random factors whereas hybrids were treated as fixed factors. The standard error of a difference (SED) was used for mean separation.

### 2.7 Combining ability effects for measured traits and parents

The ANOVA for the 96 NCD II crosses under each management condition and across the management conditions was implemented with PROC GLM in SAS using the random statement with the test option. The hybrid variance component was decomposed into males nested in sets, females nested in sets, and female × male interactions nested in sets. The male nested in sets (GCA‐male) and female nested in set (GCA‐female) together represented general combining ability (GCA), whereas the female × male interaction nested in sets represented specific combining ability (SCA) as described by Hallauer and Miranda (1988). The contribution of GCA and SCA effects of each trait was calculated using their sum of squares relative to the total sum of squares for the crosses (Singh & Chaudhary, 1985). The comparative importance of GCA or SCA effects of each trait was declared significant based on the ratio of GCA (male and female) effects to the total genotypic effects. A ratio closer to 1 indicated that GCA was a larger predictor of the trait (Baker, 1978; Machida, Derera, Tongoona, & MacRobert, 2010; Singh, Paroda, & Behl, 1986). The relative significance of maternal effects was determined through an *F* test (*P* < .05) using the GCA‐female and GCA‐male mean square variance ratios as proposed by Kearsey and Pooni (1996). The combining ability effects of inbred parents were computed as

$$\mathrm{GCA}-\mathrm{male}=X_{m}-\mu$$

$$\mathrm{GCA}-\mathrm{female}=X_{f}-\mu$$

$$\mathrm{SCA}=X_{\mathrm{mf}}-X_{m}-X_{f}+\mu$$

where *X*_m_ = the mean of hybrids that shared a common male averaged over females, environments, and replications, *X*_f _= the means of hybrids that shared a common female averaged over males, environments, and replications, *X*_mf _= the mean of a given cross, and μ = the grand mean of the experiment. The standard error was used to identify parents with significant GCA‐male and GCA‐female effects (Cox & Frey, 1984).

### 2.8 Identification of best performing single‐cross hybrids

Base indices were used to select the best performing hybrids under LN, *Striga*, HN and across the management conditions. Under LN, the low N base index proposed by Badu‐Apraku, Fakorede, Oyekunle, and Akinwale (2011) was used. *Striga*‐resistant and ‐tolerant genotypes were identified using the *Striga* resistance base index proposed by the IITA‐MIP (Badu‐Apraku & Fakorede, 2017). To identify the best‐performing hybrids across the management conditions, the multiple trait base index (MI) proposed by Badu‐Apraku et al. (2016) was adopted and the calculations were done using the data from the respective stress management conditions as follows:

$$\begin{array}{c} \mathrm{MI}=2\left(\mathrm{GY}\right)+\mathrm{EPP}-\mathrm{EA}-\mathrm{PA}-\mathrm{STYG}\\ -\mathrm{SDR}8\mathrm{WAP}-\mathrm{SDR}10\mathrm{WAP}\\ -\left(0.5\times \;\mathrm{SEC}8\mathrm{WAP}\right)-\left(0.5\times \mathrm{SEC}10\mathrm{WAP}\right) \end{array}$$

where GY = grain yield, EPP = number of ears per plant, EA = ear aspect, PA = plant aspect, STYG = stay‐green characteristic under LN, SDR8WAP = *Striga* damage rating at 8 WAP, SDR10WAP = *Striga* damage rating at 10 WAP, SEC8WAP = number of emerged *Striga* plants at 8 WAP, and SEC10WAP = number of emerged *Striga* plants at 10 WAP. The data were first standardized using PROC STDIZE in SAS to ensure that the effects of the disparity in the scales used in measuring the individual traits were curtailed.

### 2.9 Genetic distance estimation and generation of clusters using SNP markers

The SNP markers were subjected to filtering using the TASSEL software version 5.2.53 (Bradbury et al., 2007) to retain 1,701 markers with missing data <10% and minor allele frequency >5%. The pairwise frequency based genetic distance matrix was estimated using the Nei and Takezaki (1983) procedure in PowerMarker version 3.25 by Liu and Muse (2005). The Ward's minimum variance clustering procedure was used to generate the dendrogram in SAS.

### 2.10 Comparison of hybrid performance based on endosperm color and genetic distance between parental lines

The hybrids were separated into two groups based on the endosperm color of the parents from which they were generated. The hybrids generated from crosses between yellow parents only or orange parents only were grouped together as within‐group hybrids (WG), whereas those generated from yellow and orange parents were grouped together as between‐group hybrids (BG). The data were subjected to a *t* test to determine whether the differences between grain yield and genetic distances for BG and WG hybrids were significant. Pearson's phenotypic correlation was performed between genetic distance and grain yield under LN, *Striga*, HN, and across the management conditions. The *t* test and correlation analysis were executed in SAS (SAS Institute, 2012) using PROC T‐TEST and PROC CORR, respectively.

### 2.11 GGE biplot analysis

Genotype main effects and genotype × environments interaction (GGE) biplot (Yan & Kang, 2003) was used to select the high‐yielding and stable hybrids across LN, *Striga*, and HN management conditions. The GGE biplot analysis was implemented with the R software version 3.6.1 using the GGE Biplot GUI and GGE Biplot packages (http://www.rproject.org/).

## 3 RESULTS

### 3.1 Variability for grain yield and other agronomic traits

Variances for environment, genotype, and genotype × environment interaction were significant (*P* < .05 or *P* < .01) for grain yield (GY) and most measured traits under LN, HN (Table 1), *Striga*, and across the management conditions (Table 2). However, the genotype × environment interaction variances were not significant for ear aspect (EA), ear height (EHT), and husk cover (HC) under LN, and for GY and plant height (PHT) under HN conditions. Variances for GCA‐male, GCA‐female, SCA, and their environment interactions were significant (*P* < .05 or *P *< .01) for GY and most measured traits under all four conditions with few exceptions (Tables 1 and 2). The SCA variances were not significant for anthesis–silking interval (ASI) and HC under LN, HN, and *Striga*, and for ears per plant (EPP) under HN conditions. The GCA‐male × environment variances were not significant for GY, plant aspects (PA) and EA under LN, PHT under LN and HN, EHT under LN and *Striga*, HC under LN and *Striga*, DS under HN and *Striga*, and for DA under HN condition. Similarly, GCA‐female × environment variances were not significant for EHT under LN and *Striga*, PHT under LN and HN, HC under HN and *Striga*, and for DS under HN condition. The variances for SCA × environment were significant for only GY, stay‐green characteristic (STYG), EPP, and chlorophyll content under LN. However, SCA × environment were significant for GY and most traits under HN, *Striga*, and across the management condition, with few exceptions; GY, HC, PHT, and EA under HN, ASI, HC, *Striga* damage syndrome rating at 8 (SDR8WAP) and 10 (SDR10WAP) WAP, and for ASI and HC across the management conditions.

**TABLE 1:** Variances of grain yield and other traits of the extra‐early‐maturing yellow and orange quality protein maize (QPM) single‐cross hybrids under low‐N (LN) environments at Fumesua, Legon, and Nyankpala in 2018 and under five high‐N (HN) environments in 2018

Source	df	GY	DA	DS	ASI	PHT	EHT	PA	EPP	EA	HC	STYG	CC
		kg ha^−1^	d			cm		1–9^a^	no.	1–9^b^	1–5^c^	1–9^d^	
LN environments													
Env	2	47,987,446**	1,267.0**	667.6**	113.5**	73,925**	16,519**	214.9**	0.57**	66.1**	26.8**	30.7**	34,877.0**
Set	5	1,644,216**	13.3**	15.7**	2.0ns^†^	291*	267**	2.8**	0.04ns	2.0ns	0.5ns	0.7ns	69.6**
Env × Set	10	1,160,090**	5.4*	11.1**	2.4ns	201ns	57ns	1.8**	0.04ns	2.7*	0.8ns	0.4ns	46.5*
Rep (Env × Set)	15	524,688ns	3.8ns	4.7ns	1.3ns	189ns	54ns	0.4ns	0.03ns	1.0ns	0.4ns	0.5ns	15.1ns
Blk (Env × Rep)	54	1,330,978**	4.3**	6.9*	1.6ns	603**	211**	1.7**	0.05ns	4.0**	0.6ns	1.4**	30.1*
Genotype	99	1,546,389**	18.0**	25.8**	3.81**	663**	208**	2.9**	0.08**	5.6**	0.9**	1.6**	59.5**
GCA‐male (Set)	18	2,032,246**	26.4**	39.2**	5.0**	654**	176**	3.0**	0.15**	6.0**	1.0**	1.7**	56.4**
GCA‐female (Set)	18	1,905,699**	26.8**	39.7**	5.1**	1,389**	296**	5.2**	0.08*	12.3**	1.6**	2.7**	63.3**
SCA (Set)	54	760,040**	5.6**	9.8**	2.0ns	261**	99**	1.6**	0.05ns	2.5**	0.6ns	0.9**	45.1**
Genotype × Env	198	596,865**	4.2**	6.2*	2.6**	188**	62ns	1.0*	0.05**	1.6ns	0.56ns	0.9**	34.5**
GCA‐male (Set) × Env	36	531,592ns	5.0**	8.2**	3.8**	171ns	40ns	1.1ns	0.06*	1.6ns	0.5ns	0.9**	44.7**
GCA‐female (Set) × Env	36	625,435*	5.1**	7.7*	3.5**	157ns	39ns	1.1*	0.04ns	2.0*	0.6ns	0.9*	39.1**
SCA (Set) × Env	108	598,665**	3.1ns	4.3ns	1.5ns	165ns	64ns	0.7ns	0.05*	1.5ns	0.5ns	0.7*	26.1*
Error	216	374,577	2.5	4.5	1.5	129	54	0.7	0.04	1.2	0.4	0.5	19.4
HN environments													
Env	4	129,338,023**	4,895.9**	5,129.4**	48.7**	41,850**	16,300**	251.2**	0.11**	60.7**	219.3**	–	–
Set	5	22,103,320**	32.3**	24.1**	0.9ns	1,038**	1,347**	4.2**	0.08**	6.9**	1.4**	–	–
Env × Set	20	1,531,788*	1.3ns	2.3ns	1.0*	166ns	182**	0.5ns	0.02ns	0.6ns	0.5ns	–	–
Rep (Env × Set)	25	931,981ns	0.9ns	1.6ns	0.9ns	145ns	133*	1.0**	0.01ns	0.6ns	0.5ns	–	–
Blk (Env × Rep)	90	1,267,021*	3.1**	4.7**	0.9**	354**	201*	0.7**	0.02*	0.6ns	0.5*	–	–
Genotype	99	8,241,423**	29.3**	32.1**	1.5**	971**	533**	2.3**	0.06**	3.0**	0.8**	–	–
GCA‐male (Set)	18	9,106,063**	57.3**	63.6**	2.2**	1,350**	778**	2.4**	0.07**	3.3**	0.6*	–	–
GCA‐female (Set)	18	7,712,693**	44.4**	54.2**	2.8**	1,575**	652**	4.2**	0.06**	3.4**	1.2**	–	–
SCA (Set)	54	2,374,041**	3.9**	5.0**	0.8ns	294**	166**	0.8**	0.04**	1.3**	0.5ns	–	–
Genotype × Env	396	1,056,236ns	2.3**	3.43**	0.98**	132ns	119**	0.6*	0.02**	0.7**	0.5**	–	–
GCA‐male (Set) × Env	72	1,272,536*	2.0ns	2.9ns	0.9*	130ns	141**	0.5*	0.02*	1.0**	0.6**	–	–
GCA‐female (Set) × Env	72	1,343,638*	2.5*	2.9ns	1.0*	144ns	110*	0.7**	0.03**	0.9**	0.4ns	–	–
SCA (Set) × Env	216	898,243ns	2.3*	3.4*	0.9**	121ns	110**	0.5*	0.02**	0.6ns	0.4ns	–	–
Error	360	925,612	1.7	2.6	0.6	114	82	0.4	0.02	0.5	0.4	–	–

*Note*. GY, grain yield; DA, days to 50% anthesis; DS, days to 50% silking; ASI, anthesis–silking interval; PHT, plant height; EHT, ear height; PA, plant aspect; CC, chlorophyll content; EPP, ears per plant; EA, ear aspect; HC, husk cover; STYG, stay‐green characteristic; Env, environment; Rep, replication, Blk, block.

^a^ 1 denotes excellent overall phenotypic appearance, and 9 denotes extremely poor overall phenotypic appearance.

^b^ 1 denotes disease‐free, large, uniform, and well‐filled ears, and 9 denotes ears with undesirable features.

^c^ 1 denotes tightly layered husk stretching beyond the tip of the ear, and 5 denotes fully exposed ear tip.

^d^ 1 denotes hybrids displaying 0–10% dead leaf area, and 9 denotes 81–100% dead leaf area.

^*^Significant at the .05 probability level. ^**^Significant at the .01 probability level. ^†^ns, not significant.

**TABLE 2:** Variances of grain yield and other traits of the extra‐early‐maturing yellow and orange quality protein maize (QPM) single‐cross hybrids under *Striga* infestation at Nyankpala, Manga, Abuja, and Mokwa in 2018 and across 12 low‐N (LN), *Striga*, and high‐N (HN) environments in 2018

		GY	DA	DS		EPP	EA	HC	PHT	EHT	SDR8WAP	SDR10WAP	SEC8WAP	SEC10WAP
Source	df	kg ha^−1^	d		ASI	no.	1–9^a^	1–5^b^	cm		1–9^c^			
*Striga*‐infested environment														
Env	3	96,747,636.4**	1,207.59**	2,605.61**	492.69**	2.00**	31.36**	212.7**	123,227**	44,329**	28.29**	92.97**	462.42**	494.11**
Set	5	1,701,606.7**	52.82**	41.11**	4.68ns^†^	0.06ns	5.31*	1.1*	1105**	186*	4.05**	4.96**	0.41ns	0.64*
Env × Set	15	1,300,291.2**	4.81ns	9.93*	5.53ns	0.09ns	2.09ns	1.2**	194ns	80ns	0.42ns	1.06ns	0.72**	0.79**
Rep (Env × Set)	20	184,135.2ns	3.47ns	3.87ns	2.59ns	0.07ns	2.17ns	0.5ns	339**	64**	0.92ns	0.54ns	0.37ns	0.40ns
Blk (Env × Rep)	72	937,277.2**	5.84**	11.19**	5.59**	0.09**	3.07**	0.4ns	343**	112**	1.67**	2.12**	0.64ns	0.45**
Genotype	99	1,226,414**	23.99**	27.14**	5.15**	0.10**	3.37**	0.9**	857**	233**	2.63**	3.58**	0.68**	0.76**
GCA‐male (Set)	18	1,707,550.1**	35.06**	33.78**	7.36**	0.15**	2.91ns	1.2**	517**	232**	3.46**	4.82**	0.61**	0.71**
GCA‐female (Set)	18	1,666,413.3**	36.41**	41.16**	7.64**	0.12**	4.04**	1.3**	1,749**	489**	3.86**	6.33**	1.03**	1.36**
SCA (Set)	54	606,697.7**	5.58**	11.31**	2.93ns	0.08**	2.76*	0.5ns	323**	89*	1.26**	1.39*	0.57**	0.57**
Genotype × Env	297	686,069.9**	4.87**	10.96**	4.54**	0.08**	2.51**	0.5**	236**	80*	1.07**	1.46**	0.43**	0.42**
GCA‐male (Set) × Env	54	757,343.9**	4.14ns	10.9**	5.79**	0.11**	2.42ns	0.4ns	263**	76ns	1.72**	2.49**	0.45**	0.44**
GCA‐female (Set) × Env	54	634,120.5**	5.17**	13.02**	5.56**	0.07**	2.76*	0.4ns	265**	54ns	1.00*	1.26*	0.47**	0.37*
SCA (Set) × Env	162	545,667.9**	4.85**	9.87**	3.64ns	0.07**	2.43*	0.5ns	217*	88**	0.80ns	1.07ns	0.39**	0.39**
Error	287	361,350.7	3.18	5.47	3.24	0.05	1.86	0.4	161	63	0.66	0.89	0.26	0.25
Across all environments														
Env	11	454,436,973**	3,320.17**	4,560.00**	354.30**	3.03**	122.14**	167.87**	202,221.06**	72,298.80**	–	–	–	–
Set	5	19,935,938**	84.12**	68.32**	3.00ns	0.14**	11.48**	2.35**	1,823.41**	1,658.59**	–	–	–	–
Env × Set	55	1,915,285**	3.98**	7.64**	2.68**	0.05*	1.61*	0.68**	187.82*	121.37**	–	–	–	–
Rep (Env × Set)	60	585,399ns	2.39ns	3.03ns	1.57ns	0.04ns	1.31ns	0.45ns	226.48**	91.20*	–	–	–	–
Blk (Env × Rep)	216	1,173,826**	4.43**	7.58**	2.65**	0.05**	2.30**	0.49*	416.43**	175.01**	–	–	–	–
Genotype	99	7,247,086**	63.07**	69.50**	5.59**	0.13**	7.65**	1.39**	1,962.25**	732.98**	–	–	–	–
GCA‐male (Set)	18	8,052,624**	115.66**	123.73**	8.45**	0.24**	8.12**	1.70**	1,919.49**	888.56**	–	–	–	–
GCA‐female (Set)	18	8,973,498**	100.80**	122.00**	10.34**	0.14**	13.24**	2.63**	4,187.70**	1,212.94**	–	–	–	–
SCA (Set)	54	2,038,211**	8.93**	14.01**	2.10**	0.06**	3.34**	0.77**	456.37**	154.58**	–	–	–	–
Genotype × Env	1089	1,018,794**	3.61**	6.73**	2.52**	0.05**	1.60**	0.52**	191.23**	98.79**	–	–	–	–
GCA‐male (Set) × Env	198	1,207,736**	3.49**	6.88**	3.33**	0.06**	1.72**	0.51*	205.12**	105.67**	–	–	–	–
GCA‐female (Set) × Env	198	1,104,195**	3.93**	7.41**	3.02**	0.05**	1.98**	0.49*	199.75**	84.71*	–	–	–	–
SCA (Set) × Env	594	732,608**	3.11**	5.78**	1.87ns	0.05**	1.37**	0.42ns	176.92**	94.78**	–	–	–	–
Error	864	599,003	2.38	4.03	1.73	0.03	1.12	0.4	133.94	68.36	–	–	–	–

*Note*. GY , grain yield; DA, days to 50% anthesis; DS, days to 50% silking; ASI, anthesis–silking interval; EPP, ears per plant; EA , ear aspect; HC, husk cover; PHT, plant height; EHT, ear height; SDR8WAP, *Striga* damage rating at 8 wk after planting (WAP); SDR10WAP, *Striga* damage rating at 10 WAP; SEC8WAP, *Striga* emergence count at 8 WAP; SEC10WAP, *Striga* emergence count at 10 WAP; Env, environment; Rep, replication; Blk, block.

^a^ 1 denotes disease‐free, large, uniform, and well‐filled ears, and 9 denotes ears with undesirable features.

^b^ 1 denotes tightly layered husk stretching beyond the tip of the ear, and 5 denotes fully exposed ear tip.

^c^ 1 denotes normal plant growth without visible host plant damage, and 9 denotes total failure of host plant without ear formation.

^*^Significant at the .05 probability level. ^**^Significant at the .01 probability level. ^†^ns, not significant.

### 3.2 Comparative contributions of combining ability and maternal effects to grain yield and secondary traits

General combining ability effects contributed largely to the variances of GY and most traits for all management conditions with few exceptions, including chlorophyll content under LN and EA under *Striga* and HN conditions (Table 3).

**TABLE 3:** Comparative contributions of general (GCA) and specific combining ability (SCA) effects to grain yield and some secondary traits

	Low‐N condition			*Striga*‐infested condition			High‐N condition			Across management conditions		
Trait	GCA‐m	GCA‐f	SCA	GCA‐m	GCA‐f	SCA	GCA‐m	GCA‐f	SCA	GCA‐m	GCA‐f	SCA
	%											
Grain yield	32.7	30.6	36.7	32.9	32.1	35.0	38.0	32.2	29.7	34.8	38.8	26.4
Days to 50% anthesis	37.8	38.3	23.9	39.7	41.3	19.0	50.5	39.1	10.4	47.5	41.4	11.0
Days to 50% silking	36.2	36.7	27.1	31	37.8	31.2	47.9	40.8	11.3	43	42.4	14.6
Anthesis_–_silking interval	31.2	31.7	37.1	31	32.1	36.9	30	38.5	31.4	33.7	41.2	25.1
Ear aspect	23.4	47.7	28.9	19.1	26.5	54.3	13.3	23.2	63.5	25.9	42.2	32.0
Ears per plant	38.5	19.8	41.7	28.7	24	47.4	28.2	24.2	47.5	43.2	24.4	32.4
Plant aspect	23.1	39.8	37.1	–	–	–	26.8	47.1	26.1	–	–	–
Stay‐green characteristics	24.3	38.5	37.1	–	–	–	–	–	–	–	–	–
Chlorophyll content	22.1	24.8	53.1	–	–	–	–	–	–	–	–	–
*Striga* damage rating at 8 WAP^a^	–	–	–	31.2	34.8	34	–	–	–	–	–	–
*Striga* damage rating at 10 WAP	–	–	–	31.5	41.3	27.2	–	–	–	–	–	–
*Striga* emergence count at 8 WAP	–	–	–	23.2	35.8	41.1	–	–	–	–	–	–
*Striga* emergence count at 10 WAP	–	–	–	18.8	36.2	44.9	–	–	–	–	–	–

*Note*. GCA‐m and GCA‐f is the general combining ability of the male parent and female parent, respectively, and SCA is the specific combining ability.

^a^ WAP, weeks after planting.

The variations among the contributions of GCA‐male and GCA‐female effects were not significant for GY and most measured traits under LN, *Striga*, HN, and across the three management conditions except for PHT under *Striga* infestation, which displayed significant (*P* < .01) GCA‐female effects.

### 3.3 Combining ability effects of the inbred lines

Generally, the contribution of the parental lines to the crosses were not consistent across traits, individual management conditions, and across the three management conditions (Table 4). However, TZEEQI 358 displayed consistently significant (*P* < .05 or *P* < .01) and positive GCA‐male and GCA‐female effects for GY and most measured traits under LN, *Striga*, HN, and across management conditions. Contrarily, TZEEQI 396 was consistently a poor combiner for GY and most measured traits in each and across management conditions. For the LN conditions, TZEEQI 358, TZEEIORQ 9A, and TZEEQI 409 had significant and positive GCA‐male and GCA‐female effects for GY, whereas TZEEQI 353 and TZEEQI 354 displayed positive and significant GCA‐female effects. TZEEQI 372 had significant and negative GCA‐male and GCA‐female effects for STYG, whereas TZEEQI 394 and TZEEIORQ 70A exhibited significant and negative GCA‐female effects.

**TABLE 4:** General combining effects of the extra‐early‐maturing yellow and orange quality protein maize (QPM) inbred lines for grain yield and other traits in contrasting environments

	Low‐N condition				*Striga*‐infested condition						High‐N condition		Across management conditions	
	Grain yield		Stay‐green		Grain yield		SDR10WAP		SEC10WAP		Grain yield		Grain yield	
Entry	GCA‐m	GCA‐f	GCA‐m	GCA‐f	GCA‐m	GCA‐f	GCA‐m	GCA‐f	GCA‐m	GCA‐f	GCA‐m	GCA‐f	GCA‐m	GCA‐f
TZEEIORQ 46	120	−96	−0.01	0.29	603**	349*	−0.67*	−0.38*	−0.16	−0.29**	182	34	307**	107
TZEEIORQ 50	−116	−135	−0.04	−0.19	−274*	−169	0.3	0.32	−0.06	0.09	82	−213	−87	−179*
TZEEIORQ 51	−89	196	−0.02	−0.01	−204	−156	0.16	0.29	0.14	−0.03	−68	97	−119	38
TZEEIORQ 58	−94	20	0.38*	0.27	−38	366*	0.13	−0.32	−0.16	−0.32**	−112	600**	−83	377**
TZEEIORQ 63A	86	35	0.07	−0.10	−125	−24	0.22	−0.22	0.09	0.23*	−196	82	−102	35
TZEEIORQ 70A	−428**	−286*	−0.05	−0.55**	145	−183	0.15	0.11	−0.21*	−0.09	141	191	−0.2	−53
TZEEIORQ 73A	202	−152	0.15	0.37*	−72	−159	−0.17	−0.07	0.15	0.21	−147	−420*	−35	−266*
TZEEIORQ 9A	320*	418*	−0.48*	−0.08	−35	−24	−0.11	0.28	0.21*	0.19	118	−370*	118	−58
TZEEQI 353	−115	379*	0.20	0.12	156	262*	−0.11	−0.15	−0.04	−0.16	−672**	224	−257*	276*
TZEEQI 354	189	360*	0.5*	0.38*	78	36	−0.02	−0.12	0.15	0.11	−315*	312	−58	232*
TZEEQI 358	448**	440**	0.04	−0.32	285*	460**	−0.95**	−0.99**	−0.05	0.06	755**	1,071**	522**	717**
TZEEQI 361	22	15	−0.20	−0.05	353*	108	−0.4	−0.24	−0.05	−0.05	264	121	233*	90
TZEEQI 372	108	3.7	−0.47*	−0.64**	−35	159	0.01	−0.65**	0.25*	0.53**	416*	396*	188	225*
TZEEQI 374	166	−356*	−0.23	−0.02	−201	−238	0.06	−0.07	−0.04	0.07	60	−354*	−0.7	−316**
TZEEQI 392	−39	−265	−0.22	−0.10	229	10	−0.32	0.05	−0.21*	−0.05	144	81	127	−31
TZEEQI 393	−372*	182	0.25	0.29	−21	−67	0.24	0.19	0.23*	−0.03	10	266	−106	134
TZEEQI 394	‐94	−11	−0.33	−0.44*	−234	−22	0.40	−0.03	0.15	−0.04	60	−169	−77	‐80
TZEEQI 395	144	145	−0.06	−0.02	−126	221	−0.12	−0.45*	0.05	0.07	−71	43	−29	128
TZEEQI 396	−847	−899**	0.49*	1.20**	−317*	−600**	0.93**	1.54**	−0.18	−0.54**	−1,704**	−2,149**	−1,027**	−1,340**
TZEEQI 397	−55	−84	0.05	0.17	−73	−25	−0.07	0.10	−0.09	−0.03	111	‐224	8.0	−122
TZEEQI 399	6.0	−76	−0.02	0.02	84	−13	−0.37	0.07	−0.16	−0.11	−98	104	−14	120
TZEEQI 408	221	−252	−0.18	−0.29	63	−141	0.25	0.21	−0.12	0.07	158	−412*	149	−281*
TZEEQI 409	291*	456**	−0.07	−0.25	67	−18	0.02	0.11	−0.03	−0.05	533**	682**	318**	398**
TZEEQI 414	−74	−38	0.23	−0.06	−308*	−132	0.44	0.46*	0.12	0.13	349*	9	24	−50
SED	128.9	139.8	0.18	0.17	133.2	121.9	0.24	0.17	0.10	0.09	154.5	158.7	97.1	92.9

*Note*. SDR10WAP, *Striga* damage rating at 10 wk after planting (WAP); SEC10WAP, *Striga* emergence count at 10 WAP; GCA‐m , general combining ability effects of male inbred parent; GCA‐f, general combining ability effect of female inbred parent; SED, standard error of a difference.

^*^Significant at the .05 probability level. ^**^Significant at the .01 probability level.

The *Striga‐*infested management condition revealed significant and positive GCA‐male and GCA‐female effects for TZEEQI 358 and TZEEIORQ 46, TZEEIORQ 58, and TZEEQI 353 exhibited significant and positive GCA‐female effects, whereas TZEEQI 361 had significant GCA‐male effects. TZEEQI 358 had significant and negative GCA‐male and GCA‐female effects for *Striga* damage syndrome rating at 10 WAP (SDR10WAP). TZEEIORQ 46 had significant and negative GCA‐male and female effects for SDR10WAP and GCA‐female effects for number of emerged *Striga* plants at 10 WAP (SEC10WAP).

Under HN management conditions, TZEEQI 358, TZEEQI 372, and TZEEQI 409 had significant and positive GCA‐male and GCA‐female effects for GY, TZEEIORQ 58 had significant GCA‐female effects, whereas TZEEQI 414 had significant GCA‐male effects.

### 3.4 Mean grain yield performance of selected hybrids across LN, *Striga*, and HN management conditions

Using the base index, TZEEIORQ 9A × TZEEQI 397 was identified as an LN tolerant hybrid (Supplemental Table S3). However, its yield was like that of the best check (TZEEIORQ 64 × TZEEIORQ 25). Under *Striga* infestation, TZEEIORQ 58 × TZEEQI 392 was identified as *Striga* tolerant and significantly outyielded the best check, TZEEIORQ 61 × TZEEIORQ 43 by 25%. TZEEIORQ 9A × TZEEQI 354 supported the highest number of emerged *Striga* plants at both 8 (SEC8WAP) and 10 (SEC10WAP) WAP, but the GY was comparable with that of the best check. Furthermore, TZEEQI 353 × TZEEIORQ 46 had lower SEC8WAP and SEC10WAP although it was comparable with the best check. Under HN conditions, TZEEIOQR 58 × TZEEQI 392 and TZEEIORQ 58 × TZEEQI 397 were identified as the top two hybrids and yielded significantly higher than the best check by 25 and 14%, respectively. Across management conditions, the yield of the best performing hybrid, TZEEIORQ 58 × TZEEQI 392, was 28% higher than that of the best check.

### 3.5 Relationship between genetic distances and grain yield under the management conditions

The SNP markers classified the inbred lines into four clusters when 70% (*r* ^2 ^= .7) of the variation among the lines was accounted for (Supplemental Figure S1). The inbred lines were clustered mainly based on their pedigrees and the endosperm color. The yellow and orange lines were grouped distinctively into two clusters each. Wider genetic distances (significant at *P* < .01) were detected between yellow and orange endosperm lines (BG) compared with the distances between lines of the same endosperm color (WG, Table 5). The differences between BG and WG hybrid GY were significant (*P* < .01 or *p* < .05) under LN, *Striga*, HN, and across the management conditions. Genetic distances were correlated with hybrid GY under LN (*r* = .69, *P* < .01), HN (0.84, *P* < .01), across the management condition (*r* = .83, *P* < .01) and under *Striga* where a fairly weak correlation (*r* = .45, *P* < .01) was detected.

**TABLE 5:** Mean grain yield and genetic distances for within‐ and between‐endosperm color hybrids and correlations between grain yield and genetic distance

	Grain yield				
Parameter	Low N	*Striga*	High N	Across	Genetic distance
Grand mean	1,724	1,570	4,351	2,767	0.491
Maximum	2,544	2,986	6,411	4,285	0.602
Minimum	77	559	938	678	0.015
Within‐endosperm color (WG)	1,603	1,486	4,107	2,607	0.458
Between‐endosperm color (BG)	1,961	1,697	4,865	3,083	0.556
BG − WG difference	357**	211*	757**	476**	0.098**
Genetic distance ρ	.69**	.45**	.84**	.83**	

^*^Significant at the .05 probability level.

^**^Significant at the .01 probability level.

Generally, the top‐performing hybrids under LN, *Striga*, HN, and across the management conditions were crosses generated from distantly related parents that displayed higher pairwise genetic distances and were grouped under different clusters. In contrast, the worst performing hybrids were crosses developed from closely related parents with low pairwise genetic distances and grouped under the same cluster. Under LN, the best‐performing hybrid was a cross between TZEEIORQ 9A and TZEEQI 397, which were grouped under Cluster 1 and Cluster 4, respectively, and exhibited the highest pairwise genetic distance of 0.602. In contrast, the worst hybrid under LN was a cross combination involving TZEEQI 396 and TZEEQI 399 both belonging to Cluster 4 and had low pairwise genetic distance of 0.036. Under *Striga* infestation, the best hybrid was a cross between TZEEIORQ 58 and TZEEQI 392 belonging to Clusters 1 and 4, respectively, and had pairwise genetic distance of 0.534. The worst hybrid under *Striga* was generated from a cross between TZEEQI 397 and TZEEQI 396 both belonging to Cluster 4 and had low pairwise genetic distance of 0.024. Under the HN environment, the best‐performing hybrid was derived from a cross between TZEEIORQ 58 and TZEEQI 397, which were grouped under Clusters 1 and 4, respectively. The worst hybrid under HN condition was a cross between TZEEQI 396 and TZEEQI 393 both belonging to Cluster 4 and had the lowest pairwise genetic distance of 0.015.

### 3.6 Genotype × environment interaction and stability of the hybrids

**FIGURE 1:**
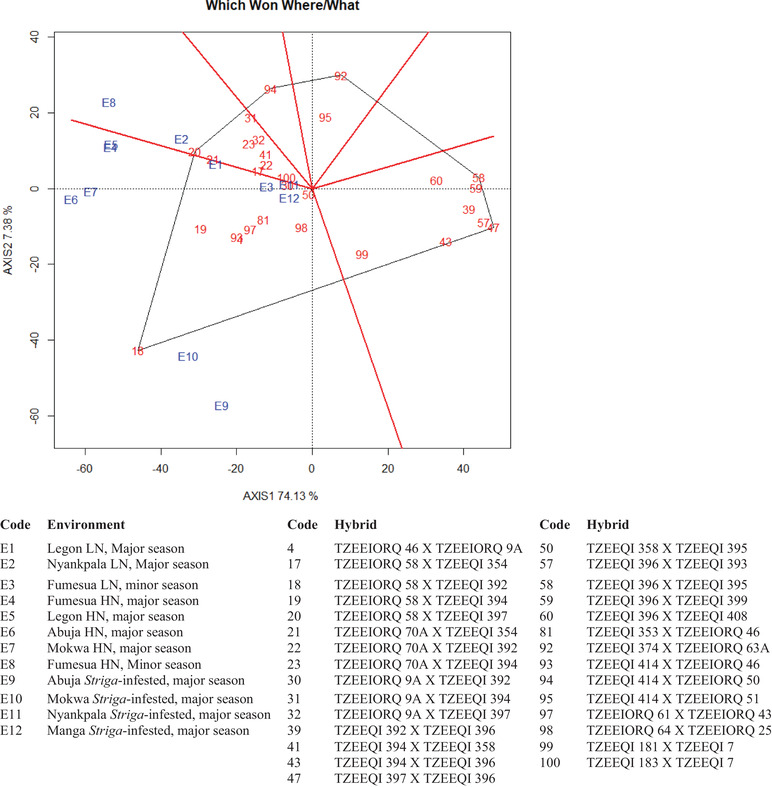
A “which‐won‐where/what” genotype and genotype × environment (GGE) biplot of the 29 (best 15, worst 10, and 4 checks) hybrids across low‐N (LN), *Striga*, and high N (HN) environments in Ghana and Nigeria during the major and minor growing seasons, 2018

**FIGURE 2:**
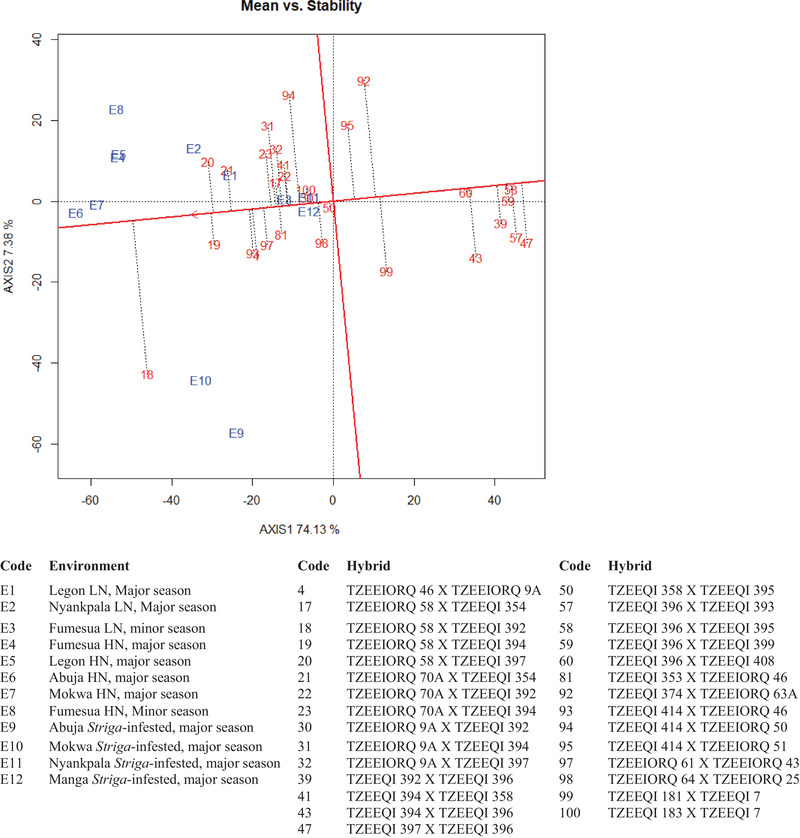
An entry/tester genotype and genotype × environment (GGE) biplot of 29 (best 15, worst 10, and 4 checks) extra‐early quality protein maize (QPM) hybrids evaluated across low‐N (LN), *Striga*, and high N (HN) environments in Ghana and Nigeria during the major and minor growing season, 2018

The mean GY of 29 hybrids (best 15, bottom 10, and four checks) selected across the 12 environments using the multiple‐stress base index were used to generate GGE biplots. The first and second principal components (PC1 and PC2) axes collectively explained 81.5% of the total variance for GY. The “which‐won‐where/what” polygon view grouped the environments into two mega‐environments with each having a wining hybrid (Figure 1). Ten environments (E1, E3, E4, E5, E6, E7, E9, E10, E11, and E12) were grouped together with hybrid 18 (TZEEIORQ 58 × TZEEQI 392) as the wining genotype. The second mega‐environment grouped E2 and E8 together with hybrid 20 (TZEEIORQ 58 × TZEEQI 397) as the winning genotype. Hybrids 47 (TZEEQI 397 × TZEEQI 396) and 58 (TZEEQI 396 × TZEEQI 395) were the worst genotypes across environments. The ”mean vs. stability” biplot enabled the identification of hybrids with high GY and stability across the 12 environments (Figure 2). Hybrids with longer projection unto the vertical axis on the left side were considered as highest yielding, whereas those situated closer to the horizontal axis were the considered as most stable. Based on these criteria, hybrid 18 (TZEEIORQ 58 × TZEEQI 392), 19 (TZEEIORQ 58 × TZEEQI 394) and 20 (TZEEIORQ 58 × TZEEQI 397) were the top three high‐yielding hybrids. However, 19 (TZEEIORQ 58 × TZEEQI 394) and 20 (TZEEIORQ 58 × TZEEQI 397) were more stable compared with 18 (TZEEIORQ 58 × TZEEQI 392).

## 4 DISCUSSION

The significant variations present among the hybrids for GY and other measured traits under each and across the management conditions was an indication of genetic variability and implied that selecting for important traits could help achieve desirable genetic gains (Ertiro et al., 2017; Oyekale et al., 2020). The significant variations observed for environments under each and across the three management conditions suggested that the environments were remarkably different. These differences could be attributed to the variable soil types, rainfall patterns, temperatures, and solar radiation intensity and durations at the contrasting experimental sites and countries used in the study. The result agreed with the findings of others (Annor & Badu‐Apraku, 2016; Obeng‐Bio et al., 2019). In this study, the genotype × environment interaction variances for GY were significant under LN, *Striga*, and across the three management conditions but were not significant under HN conditions. This implied that the rankings in GY of the hybrids were not consistent under LN, *Striga*, and across the three management. The rankings in GY of the hybrids were, however, similar under the HN management leading to the nonsignificant genotype × environment interaction variances detected. This suggested that the performance of the hybrids under HN was largely influenced by their genetic potential with little environmental influence, and the change in the ranking of genotypes in the different environments was not significant. The results indicated that the hybrids had wide adaptations to different environments under HN conditions. The findings of this study affirmed the importance of environments in identifying promising genotypes and the need for multilocation trials in hybrid breeding programs (Badu‐Apraku et al., 2016; Yan, Kang, Ma, Woods, & Cornelius, 2007)

A clear understanding of the amount of additive and dominance gene action regulating GY and the secondary traits in inbred lines is a prerequisite in setting breeding strategies for improving target traits (Ertiro et al., 2017; Zhou et al., 2018). The present study detected significant GCA and SCA variances for GY and other important traits under the three and across the management conditions, suggesting the contribution of both additive and nonadditive genetic effects. However, the superiority of GCA over SCA for GY and the majority of the other measured traits indicated that additive gene action played a larger role. This result implied that the parents that demonstrated significant and positive GCA effects for GY could produce superior hybrids when used in hybrid combinations. Also, populations developed from the crosses between such parents could accumulate high doses of beneficial alleles for tolerance to the targeted stresses from which desirable inbred lines could be extracted. This is because traits controlled by additive gene action are highly heritable, have less environmental influence, and are more amenable to improvement through recurrent selection schemes (Chigeza, Mashingaidze, & Shanahan, 2014; Ertiro et al., 2017). The results further suggested that the use of recurrent selection methods in the improvement of all traits except chlorophyll content would be successful. However, production of hybrids with high chlorophyll content would be effective using this set of inbred lines. The results of the present study were in agreement with that of Badu‐Apraku et al. (2015), Ertiro et al. (2017), and Obeng‐Bio et al. (2019), who reported preponderance of additive genes for GY under LN management conditions. The results were, however, inconsistent with the reports of others (Badu‐Apraku et al., 2016; Mafouasson et al., 2017; Makumbi et al., 2011), who detected the predominance of nonadditive gene action for GY under LN conditions. The superior additive gene action observed for GY under *Striga* infestation confirmed the reports of Ifie et al. (2015) but were inconsistent with the finding of Badu‐Apraku et al. (2016) and Annor et al. (2019), who found predominance of nonadditive gene action. The preponderant additive gene action detected for *Striga* damage and the number of emerged *Striga* plants at 8 and 10 WAP are in agreement with the results of other researchers (Ifie et al., 2015; Yallou et al., 2009). The results, however, are not consistent with the findings of Badu‐Apraku et al. (2016), who reported nonadditive gene action for *Striga* damage and number of emerged *Striga* plants. The results are also inconsistent with those of Badu‐Apraku, Menkir, and Lum (2007), who reported that additive gene action controlled the inheritance of *Striga* damage, whereas nonadditive gene action conditioned the inheritance of the number of emerged *Striga* plants. The predominance of additive gene action detected for GY and other traits under HN conditions in this study is in agreement with the results of other researchers (Annor et al., 2019; Ertiro et al., 2017; Obeng‐Bio et al., 2019) but is inconsistent with the findings of Fan et al. (2014), who reported nonadditive gene action. Although the inbred lines used for this study were extracted from *Striga*‐resistant populations, the prominence of the additive gene action for GY and most measured traits was not limited to the *Striga* management conditions. The preponderance of the additive gene action over the nonadditive under LN conditions could be attributed to the reported concomitant improvement in LN tolerance associated with the selection for *Striga* resistant genotypes under 30 kg N ha^−1^ (Ifie et al., 2015). The greater additive genetic effects observed for most studied traits relative to the nonadditive could be attributed to the genetic material, the environments, or test locations and intensity levels of the stresses imposed in this study. Also, the use of QPM germplasm could have influenced the inheritance patterns observed for GY and other traits. The predominance of SCA effects observed for chlorophyll content under LN management conditions indicated that chlorophyll content was under nonadditive gene control (e.g., dominance and epistasis), implying that inbred lines that produced hybrids with high chlorophyll content should be considered for improvement of the trait.

The nonsignificant GCA‐female variance ratio for GY and most measured traits under the various management conditions implied that maternal influences did not moderate the inheritance pattern. On the contrary, significant GCA‐female effects for plant height under *Striga* infestation suggested that maternal genes moderated the inheritance of plant height. Therefore, inbred lines that displayed significant and positive GCA‐female effects for plant height under *Striga*‐infested management conditions should not be used as female parents in crosses in order to limit the transfer of genes for tall plants to their progenies. Tall plants are not desirable in hybrid production due to their tendency to lodge. The results of the present study agreed with those of other researchers (Badu‐Apraku et al., 2016; Ifie et al., 2015; Obeng‐Bio et al., 2019) who documented nonsignificant maternal effects for GY under contrasting environments but contradicted the findings of Oyekale et al. (2020), who reported significant maternal effects for GY under LN, *Striga*, and HN conditions.

The significant GCA‐male × environment, GCA‐female × environment, and SCA × environment interactions for GY under the different management conditions suggested that the combining ability of the parents varied across LN, *Striga*, and HN conditions. The result inferred that the additive and nonadditive gene effects interacted with the environments in their expression, which justified the importance of multi‐location testing in breeding LN tolerant and *Striga‐*resistant and ‐tolerant hybrids.

The potential value of an inbred line in a breeding program is determined by the GCA effects of the line and the SCA effects of its hybrid (Ertiro et al., 2017). Inbred lines with good GCA effects contribute invaluably to breeding programs, as they serve as parents for superior hybrid development. They are also good sources of favourable alleles for population improvement using recurrent selection or inbred recycling and are potential testers for evaluating newly developed inbred lines (Makumbi et al., 2011). TZEEQI 358, which displayed significant GCA male and female values for GY and the majority of the measured traits under LN, *Striga*, and across the management conditions, would transfer the favorable alleles to its progenies for improvement of target traits. Similar inferences could be drawn for TZEEIORQ 9A and TZEEQI 409 under LN, TZEEIORQ 46 under *Striga* infestation, TZEEQI 372 and TZEEQI 409 under HN, and TZEEQI 361 and TZEEQI 409 across the management conditions. The significance of the GCA‐ female variances of GY for TZEEQI 354 under LN, TZEEIORQ 58 and TZEEQI 353 under *Striga*, TZEEIORQ 58 under HN, and TZEEIORQ 58, TZEEQI 353, TZEEQI 354, and TZEEQI 372 across environments indicated that they have the potential to transfer favorable alleles for the improvement of their progenies when used as female parents. Similar deductions are applicable for TZEEQI 361 under *Striga*, TZEEQI 414 under HN, and TZEEIORQ 46 across the management conditions when used as male parents due to the significant GCA‐male effects for GY. The significant and negative GCA‐male and GCA‐female effects of TZEEQI 372 for STYG under LN indicated that the inbred line possessed favorable alleles for delayed leaf senescence and can transmit this trait to its progenies. The significant negative GCA‐male effect of TZEEIORQ 46 for SDR8WAP and SDR10WAP, and GCA‐female effects for SEC8WAP and SEC10WAP, indicated that the inbred lines possessed favorable alleles for *Striga* resistance. Thus, progenies from crosses involving TZEEIORQ 46 would be characterized by reduced *Striga* emergence and host damage syndrome rating.

The base indices used in this study identified hybrids that significantly outyielded the best check under the stress and nonstress environments except under LN where the GY of the best hybrids was comparable with that of best check. The yield of the top‐performing hybrid TZEEIORQ 58 × TZEEQI 392 across test environments was significantly higher than that of the best check. This indicated that TZEEIORQ 58 × TZEEQI 392 was superior in tolerance to LN and resistance to *Striga* and had less yield reduction under the stresses compared with the best check. The hybrid could potentially increase maize production in LN and *Striga* endemic production agroecologies of SSA. In *Striga* research, resistance to *Striga* implies the ability of the host plant to stimulate the germination of *Striga* seeds but prevent the attachment of the parasite to its roots or kill the attached parasite. The resistant plant supports significantly fewer *Striga* plants and produces a higher yield than a susceptible genotype (Ejeta, Butler, & Babiker, 1992; Haussmann et al., 2000; Rodenburg, Bastiaans, & Kropff, 2006). Contrarily, a *Striga*‐tolerant genotype germinates and supports as many *Striga* plants as the intolerant genotype but produces more grain and stover and displays fewer damage symptoms (Kim, 1994). The best hybrid under *Striga* infestation outyielded the best hybrid check by 25%, indicating that it was more resistant to *Striga* than the check. TZEEIORQ 9A × TZEEQI 354 was identified as *Striga* tolerant because it had reduced *Striga* damage despite supporting the growth of the highest number of *Striga* plants. TZEEQI 353 × TZEEIORQ 46 was also identified as resistant because it supported fewer emerged *Striga* plants. TZEEIORQ 9A × TZEEQI 354 and TZEEQI 353 × TZEEIORQ 46 could potentially boost maize production in *Striga* endemic agroecologies of SSA. Under HN conditions, TZEEIOQR 58 × TZEEQI 392 and TZEEIORQ 58 × TZEEQI 397 which significantly outyielded the best hybrid check could contribute to increased maize yield under high fertilizer input and *Striga*‐free growing conditions. The best hybrids identified for each management condition and across the environments can potentially address the protein deficiency and malnutrition challenges in SSA. However, nutritional assay of tryptophan, lysine, and carotenoid content would be required to determine whether the levels meet the minimum body requirements.

In this study, the top‐performing hybrids under the contrasting management conditions were crosses involving orange and yellow parental lines. This was not surprising because the yellow and orange lines were extracted from different populations, had wider genetic distances, and were therefore expected to exhibit high heterosis compared with the crosses involving inbred lines of the same endosperm color. The close genetic relatedness among the parents of the worst‐performing hybrids possibly underlined their poor GY under both the stress and nonstress conditions. The parents of most of the worst hybrids had both yellow parents with similar pedigrees; hence, they had lower genetic distances resulting in lower heterosis. This pattern could possibly be attributed to the observed significant correlation between GY and genetic distances under the three management conditions. The result inferred that the development of superior hybrids using the inbred lines would require selection of parents with high genetic distances for maximum heterosis (Govindaraj, Vetriventhan, & Srinivasan, 2015; Nyombayire, Derera, Sibiya, Gasura, & Ngaboyisonga, 2016).

The GGE biplot analysis identified high‐yielding and stable hybrids across the LN, *Striga*, and HN environments. Genotype × environment interaction is useful in identifying genotypes with broad adaptation to different environments and also for selecting location‐specific genotypes (Badu‐Apraku & Fakorede, 2017). Hybrids with vast adaptation to the production environments are the most appropriate for farmers in SSA due to the wide environmental variations experienced across locations and seasons. In the present study, the “which‐won‐where/what” biplot was used to select location specific hybrids, whereas the “mean vs. stability” biplot identified hybrids that were stable under LN, *Striga*, and HN conditions. In the “which‐won‐where/what” biplot, one hybrid (TZEEIORQ 58 × TZEEQI 392) was identified as the winning hybrid in 10 environments—that is, LN at Legon and Fumesua (minor), *Striga* at Abuja, Mokwa, Nyankpala, and Manga and HN conditions at Legon, Abuja, Mokwa, and Fumesua (major seasons). These 10 environments represented one mega‐environment, since they were grouped together and had hybrid 18 (TZEEIORQ 58 × TZEEQI 392) as the winning hybrid. The results suggested that the performance of a genotype in any one of these environments could predict its performance in the other nine environments. Environments E2 (Nyankpala LN, major season) and E8 (Fumesua HN, minor season) were also grouped as one mega‐environment with hybrid 20 (TZEEIORQ 58 × TZEEQI 397) as the winning genotype. This suggested that TZEEIORQ 58 × TZEEQI 397 would produce superior yield under HN growing conditions in Fumesua (minor season) and would suffer less yield penalty under LN at Nyankpala. The “mean vs. stability” biplot identified hybrid 18 (TZEEIORQ 58 × TZEEQI 392) to be characterized by high GY but low stability across the 12 environments. This is ascribable to the large differences detected between GY in the stress and nonstress environments. Hybrids 19 (TZEEIORQ 58 × TZEEQI 394) and 20 (TZEEIORQ 58 × TZEEQI 397) were characterized by high GY and stability across the test environments. TZEEIORQ 58 × TZEEQI 392 is therefore recommended for location‐specific plantings, particularly in *Striga*‐prone environments at Abuja (E9) and Mokwa (E10). In contrast, TZEEIORQ 58 × TZEEQI 397 was characterized by high GY and stability across test environments and has broad adaptation. Hence, it is recommended for production under LN, *Striga*, and HN growing conditions in Ghana, Nigeria, and other environments in the SSA region where similar agroecologies exist.

## 5 CONCLUSION

The inheritance of grain yield and most measured traits under LN, *Striga* infestation, HN, and across the management conditions was largely regulated by additive gene effects relative to the nonadditive gene effects. However, chlorophyll content under LN was largely influenced by nonadditive gene effects. The inheritance of grain yield and most measured traits was not influenced by maternal effects except for plant height under *Striga* infestation. TZEEQI 358 displayed significant and positive GCA‐male and GCA‐female effects for grain yield and most agronomic traits under the LN, *Striga* infestation, HN, and across the management conditions. The SNP‐based genetic distances were associated with grain yield performance under LN, *Striga* infestation, HN, and across the management conditions. TZEEIORQ 58 × TZEEQI 392 was the best‐performing hybrid across locations but was least stable across the environments. TZEEIORQ 58 × TZEEQI 397 was high yielding and the most stable hybrid across the LN, *Striga‐*infested, and HN environments.

## AUTHOR CONTRIBUTIONS

P. Abu, B. Badu‐Apraku, B. E. Ifie, P. Tongoona, and S. K. Offei designed this study; B. Badu‐Apraku developed the genetic materials used in the study; P. Abu and B. Badu‐Apraku conducted the experiments; P. Abu analyzed the data; P. Abu prepared the draft manuscript; all authors revised the manuscript.

## FUNDING

Financial support for this research was provided by the USAID and DAAD through WACCI, which sponsored the first author for her PhD studies and the DTMA/STMA funding by the Bill and Melinda Gates Foundation (Grant/Award no.: OPP1134248).

## CONFLICT OF INTEREST

The authors declare no conflict of interest

## Supporting information

Supplemental Table S1. Description of the 24 lines used in the present study
Supplemental Table S2. Description of the 12 environments used for the present study
Supplemental Table S3. Performance of top 15 and bottom 9 hybrids selected based on grain yield and some secondary traits using the multiple trait base index across LN, *Striga*, and HN environments in Ghana and Nigeria
Supplemental Figure S1. Dendrogram showing the genetic relationship among the 24 inbred parents with SNP markers using Nei 1983 genetic distance methods and the Ward's minimum variance clustering method.

## Data Availability

Data pertaining to this study are available to authorized users at the International Institute of Tropical Agriculture (IITA) maize improvement program data repository.
