# Supplementary material for: Genetics of extra‐early‐maturing yellow and orange quality protein maize inbreds and derived hybrids under low soil nitrogen and Striga infestation
Source: Crop Sci. 2020 Dec 22;61(2):1052–72. doi: 10.1002/csc2.20384 (PMC8048537; doi:10.1002/csc2.20384)
Supplement: Supplementary file 1 — Supplemental Table S1. Description of the 24 lines used in the present study Supplemental Table S2. Description of the 12 environments used for the present study Supplemental Table S3. Performance of top 15 and bottom 9 hybrids selected based on grain yield and some secondary traits using the multiple trait base index across LN, Striga, and HN environments in Ghana and Nigeria Supplemental Figure S1. Dendrogram showing the genetic relationship among the 24 inbred parents with SNP markers using Nei 1983 genetic distance methods and the Ward's minimum variance clustering method. [file CSC2-61-1052-s001.docx]

Supplementary Table 1. Description of the twenty-four lines used in the present study

| **S/N** | **Inbred** | **Grain colour** | **Pedigree** | **Tryptophan (%)** | **Reaction to**  **LN** | **Reaction to *Striga*** | **Set** |
| --- | --- | --- | --- | --- | --- | --- | --- |
| 1 | TZEEIORQ 46 | Orange | 2009 TZEE-OR2 STR QPM S6 22-3/3-3/3-1/3-3/3-3/3 | 0.08 | Tolerant | Tolerant | 1 |
| 2 | TZEEIORQ 50 | Orange | 2009 TZEE-OR2 STR QPM S6 27-1/5-1/3-1/3-1/2-1/2 | 0.08 | Susceptible | Susceptible | 1 |
| 3 | TZEEIORQ 51 | Orange | 2009 TZEE-OR2 STR QPM S6 27-1/5-1/3-1/3-2/2-2/2 | 0.07 | Susceptible | Susceptible | 1 |
| 4 | TZEEIORQ 63A | Orange | 2009 TZEE-OR2 STR QPM S6 27A-5/5-2/2-1/2-1/2-1/2 | 0.07 | Tolerant | Susceptible | 1 |
| 5 | TZEEIORQ 58 | Orange | 2009 TZEE-OR2 STR QPM S6 27-3/5-1/3-1/2-1/3-1/2 | 0.08 | Susceptible | Tolerant | 2 |
| 6 | TZEEIORQ 70A | Orange | 2009 TZEE-OR2 STR QPM S6 34-1/1-3/3-1/1-4/4-2/2A | 0.08 | Susceptible | Tolerant | 2 |
| 7 | TZEEIORQ 73A | Orange | 2009 TZEE-OR2 STR QPM S6 58-1/1-1/2-1/1-3/3-1/2A | 0.08 | Tolerant | Tolerant | 2 |
| 8 | TZEEIORQ 9A | Orange | 2009 TZEE-OR2 STR QPM S6 15-1/2-3/3-4/4-1/2-1/1A | 0.08 | Susceptible | Susceptible | 2 |
| 9 | TZEEQI 354 | Yellow | 99 TZEE-Y POP STR QPM S7 Inb. 32B-2/2-1/1-1/1-1/2-2/2 | 0.08 | Susceptible | Susceptible | 3 |
| 10 | TZEEQI 392 | Yellow | 99 TZEE-Y POP STR QPM S7 Inb. 233-2/2-1/4-1/2-3/3-1/8 | 0.07 | Tolerant | Susceptible | 3 |
| 11 | TZEEQI 394 | Yellow | 99 TZEE-Y POP STR QPM S7 Inb. 233-2/2-1/4-2/2-1/1-1/1 | 0.07 | Tolerant | Susceptible | 3 |
| 12 | TZEEQI 397 | Yellow | 99 TZEE-Y POP STR QPM S7 Inb. 233-2/2-2/4-1/2-1/3-1/6 | 0.07 | Tolerant | Tolerant | 3 |
| 13 | TZEEQI 358 | Yellow | 99 TZEE-Y POP STR QPM S7 Inb. 83-3/4-1/4-2/2-1/2-3/3 | 0.07 | Tolerant | Susceptible | 4 |
| 14 | TZEEQI 372 | Yellow | 99 TZEE-Y POP STR QPM S7 Inb. 161-4/4-1/5-2/2-2/2-1/3 | 0.07 | Tolerant | Susceptible | 4 |
| 15 | TZEEQI 396 | Yellow | 99 TZEE-Y POP STR QPM S7 Inb. 233-2/2-2/4-1/1-1/1-4/6 | 0.08 | Tolerant | Susceptible | 4 |
| 16 | TZEEQI 409 | Yellow | 99 TZEE-Y POP STR QPM S8 Inb. 22-1/1-3/3-2/2-1/1-1/2-1/2 | 0.07 | Susceptible | Tolerant | 4 |
| 17 | TZEEQI 393 | Yellow | 99 TZEE-Y POP STR QPM S7 Inb. 233-2/2-3/4-1/2-1/3-1/1 | 0.07 | Tolerant | Susceptible | 5 |
| 18 | TZEEQI 395 | Yellow | 99 TZEE-Y POP STR QPM S7 Inb. 233-2/2-2/4-1/1-1/1-2/6 | 0.07 | Tolerant | Tolerant | 5 |
| 19 | TZEEQI 399 | Yellow | 99 TZEE-Y POP STR QPM S7 Inb. 233-2/2-3/4-1/2-3/3-9/9 | 0.07 | Tolerant | Susceptible | 5 |
| 20 | TZEEQI 408 | Yellow | 99 TZEE-Y POP STR QPM S7 Inb. 233-2/2-3/4-2/2-2/2-1/6 | 0.08 | Tolerant | Susceptible | 5 |
| 21 | TZEEQI 353 | Yellow | 99 TZEE-Y POP STR QPM S7 Inb. 29B-2/2-2/3-2/2-1/1-2/3 | 0.07 | Susceptible | Susceptible | 6 |
| 22 | TZEEQI 361 | Yellow | 99 TZEE-Y POP STR QPM S7 Inb. 83-3/4-1/4-2/2-2/2-2/3 | 0.08 | Tolerant | Tolerant | 6 |
| 23 | TZEEQI 374 | Yellow | 99 TZEE-Y POP STR QPM S7 Inb. 154-1/2-4/4-2/2-2/2-1/5 | 0.07 | Susceptible | Tolerant | 6 |
| 24 | TZEEQI 414 | Yellow | 99 TZEE-Y POP STR QPM S8 Inb. 154-1/2-4/4-2/2-2/2-1/1-3/3 | 0.09 | Susceptible | Susceptible | 6 |
|  |  |  |  |  |  |  |  |

**Supplementary Table 2. Description of the 12 environments used for the present study**

| **Environment** | **Country** | **Location** | **Location Coordinates** | **Annual**  **rainfall**  **(mm)** | **Altitude (m)** | **Planting Season** | **Treatment** |
| --- | --- | --- | --- | --- | --- | --- | --- |
| 1 | Ghana | Fumesua | 6°41′ N, 1°28′ W | 1500 | 286 | Minor | LN |
| 2 | Ghana | Fumesua | 6°41′ N, 1°28′ W | 1500 | 286 | Major | HN |
| 3 | Ghana | Fumesua | 6°41′ N, 1°28′ W | 1500 | 286 | Minor | HN |
| 4 | Ghana | Legon | 5°38′ N and 00°11′ E | 809 | 97 | Major | LN |
| 5 | Ghana | Legon | 5°38′ N and 00°11′ E | 809 | 97 | Major | HN |
| 6 | Ghana | Nyankpala | 9°4’N and 0°98’W | 1,100 | 183 | Major | LN |
| 7 | Ghana | Nyankpala | 9°4’N and 0°98’W | 1,100 | 183 | Major | *Striga*-infested |
| 8 | Ghana | Manga | 11°01′ N, 0°16' W | 921 | 252 | Major | *Striga*-infested |
| 9 | Nigeria | Abuja | 9°15′ N and 7° 20′ E | 1,700 | 300 | Major | *Striga*-infested |
| 10 | Nigeria | Abuja | 9°15′ N and 7° 20′ E | 1,700 | 300 | Major | HN |
| 11 | Nigeria | Mokwa | 9°18′ N and 5°04′ E | 1,100 | 457 | Major | *Striga*-infested |
| 12 | Nigeria | Mokwa | 9°18′ N and 5°04′ E | 1,100 | 457 | Major | HN |

LN = low nitrogen, HN = high nitrogen

**Supplementary Table 3**. Performance of top 15 and bottom 9 hybrids selected based on grain yield and some secondary traits using the multiple trait base index across LN, *Striga* and HN environments in Ghana and Nigeria

|  | Grain yield | | | | EPP | EA (1-9) | PA (1-9) | | STYG  (1-9) |  | |  | |  |
| --- | --- | --- | --- | --- | --- | --- | --- | --- | --- | --- | --- | --- | --- | --- |
| HYBRID | LN | *Striga* | HN | Across |  |  |  |  |  | SDR8 WAP | SDR10 WAP | SEC8 WAP | SEC10 WAP | MI |
| TZEEIORQ 58 X TZEEQI 392 | 2476 | 2986 | 6411 | 4285 | 0.92 | 4.2 | | 3.6 | 4.2 | 4.4 | 4.5 | 9.5 | 7.5 | 11.2 |
| TZEEIORQ 61 X TZEEIORQ 43* | 1671 | 2386 | 5141 | 3355 | 0.94 | 4.2 | | 3.9 | 3.4 | 3.9 | 4.6 | 7.5 | 7.9 | 9.4 |
| TZEEQI 353 X TZEEIORQ 46 | 2030 | 2214 | 4674 | 3193 | 0.93 | 4.1 | | 3.5 | 4.1 | 4.3 | 5.1 | 6.0 | 6.7 | 7.4 |
| TZEEIORQ 58 X TZEEQI 397 | 2348 | 1711 | 5918 | 3623 | 0.95 | 4.2 | | 3.3 | 4.5 | 5.0 | 5.2 | 7.7 | 6.5 | 7.2 |
| TZEEIORQ 70A X TZEEQI 354 | 2414 | 1773 | 5752 | 3592 | 0.89 | 3.8 | | 3.4 | 4.1 | 4.7 | 5.3 | 17.1 | 16.2 | 6.3 |
| TZEEQI 414 X TZEEIORQ 46 | 2519 | 2277 | 4895 | 3429 | 0.90 | 4.7 | | 3.6 | 4.5 | 4.0 | 4.9 | 3.4 | 12.1 | 6.0 |
| TZEEQI 358 X TZEEQI 395 | 1633 | 2013 | 3867 | 2690 | 0.94 | 4.1 | | 3.2 | 3.6 | 3.9 | 4.4 | 13.3 | 15.4 | 5.7 |
| TZEEIORQ 70A X TZEEQI 394 | 1719 | 1475 | 5387 | 3166 | 0.88 | 4.0 | | 3.2 | 3.4 | 4.8 | 5.9 | 12.9 | 9.0 | 5.7 |
| TZEEIORQ 58 X TZEEQI 354 | 1720 | 1951 | 4978 | 3154 | 0.92 | 4.3 | | 3.5 | 4.8 | 4.1 | 4.6 | 10.7 | 11.9 | 5.7 |
| TZEEIORQ 64 X TZEEIORQ 25* | 2200 | 1763 | 3994 | 2803 | 0.97 | 4.3 | | 3.8 | 3.6 | 4.5 | 4.9 | 6.3 | 10.2 | 5.6 |
| TZEEIORQ 58 X TZEEQI 394 | 1558 | 2318 | 5778 | 3569 | 0.88 | 4.3 | | 3.9 | 4.1 | 4.6 | 5.2 | 9.7 | 11.3 | 5.2 |
| TZEEIORQ 9A X TZEEQI 397 | 2418 | 1846 | 5136 | 3359 | 0.96 | 4.3 | | 3.5 | 4.1 | 4.9 | 5.3 | 20.7 | 18.8 | 4.9 |
| TZEEIORQ 46 X TZEEIORQ 70A | 906 | 2238 | 5235 | 3154 | 0.72 | 4.7 | | 3.5 | 3.7 | 3.9 | 5.1 | 9.2 | 11.1 | 4.7 |
| TZEEIORQ 9A X TZEEQI 354 | 2541 | 2346 | 4552 | 3314 | 1.01 | 4.3 | | 3.7 | 4.8 | 4.7 | 5.4 | 20.5 | 23.4 | 4.2 |
| TZEEIORQ 9A X TZEEQI 394 | 2503 | 1347 | 5006 | 3160 | 0.81 | 4.1 | | 3.7 | 3.5 | 5.7 | 6.2 | 23.7 | 24.1 | 0.5 |
| TZEEQI 183 X TZEEQI 7* | 2487 | 1721 | 4044 | 2880 | 0.85 | 4.8 | | 4.1 | 4.4 | 5.2 | 5.8 | 12.9 | 16.0 | -1.1 |
| TZEEQI 181 X TZEEQI 7* | 879 | 1675 | 3341 | 2170 | 0.78 | 5.1 | | 4.4 | 4.0 | 5.0 | 5.8 | 15.6 | 22.7 | -4.8 |
| TZEEQI 394 X TZEEQI 396 | 626 | 1499 | 2040 | 1506 | 0.69 | 5.9 | | 5.3 | 4.3 | 6.0 | 6.7 | 11.0 | 9.5 | -12.6 |
| TZEEQI 392 X TZEEQI 396 | 175 | 986 | 1528 | 1009 | 0.66 | 6.5 | | 5.5 | 5.2 | 5.7 | 6.4 | 3.6 | 8.4 | -16.0 |
| TZEEQI 396 X TZEEQI 395 | 638 | 559 | 1247 | 865 | 0.80 | 6.3 | | 5.8 | 5.9 | 6.3 | 7.2 | 6.5 | 5.9 | -18.2 |
| TZEEQI 396 X TZEEQI 408 | 932 | 800 | 1777 | 1240 | 0.61 | 6.6 | | 5.6 | 5.1 | 7.0 | 7.9 | 4.5 | 8.7 | -19.5 |
| TZEEQI 396 X TZEEQI 393 | 134 | 755 | 1103 | 678 | 0.68 | 6.4 | | 5.7 | 5.5 | 6.7 | 7.7 | 5.0 | 6.0 | -20.6 |
| TZEEQI 396 X TZEEQI 399 | 77 | 833 | 1075 | 790 | 0.70 | 6.9 | | 5.8 | 6.0 | 6.5 | 7.3 | 6.1 | 7.1 | -21.3 |
| TZEEQI 397 X TZEEQI 396 | 604 | 721 | 938 | 782 | 0.50 | 6.9 | | 5.9 | 5.5 | 6.9 | 8.2 | 9.8 | 9.2 | -25.5 |
| Mean | 1724 | 1570 | 4351 | 2767 | 0.85 | 4.73 | | 4.83 | 4.17 | 4.93 | 5.61 | 13.06 | 14.51 |  |
| *S.E.D.* | 355.2 | 403.88 | 591.85 | 498.36 | 4.57 | 0.5 | | 0.48 | 0.4 | 0.46 | 0.58 | 5.71 | 6.18 |  |

*=check, EPP=ears per plant, EA=ear aspect, PA=plant aspect, STYG=stay-green characteristics, SDR8WAP=*Striga* damage rating at 8 WAP, SDR10WAP=*Striga* damage rating at 8 WAP SEC8WAP=*Striga* emergence count at 8 WAP, SEC10WAP=*Striga* emergence count at 10 WAP MI=multiple traits base index.

Supplementary Figures:


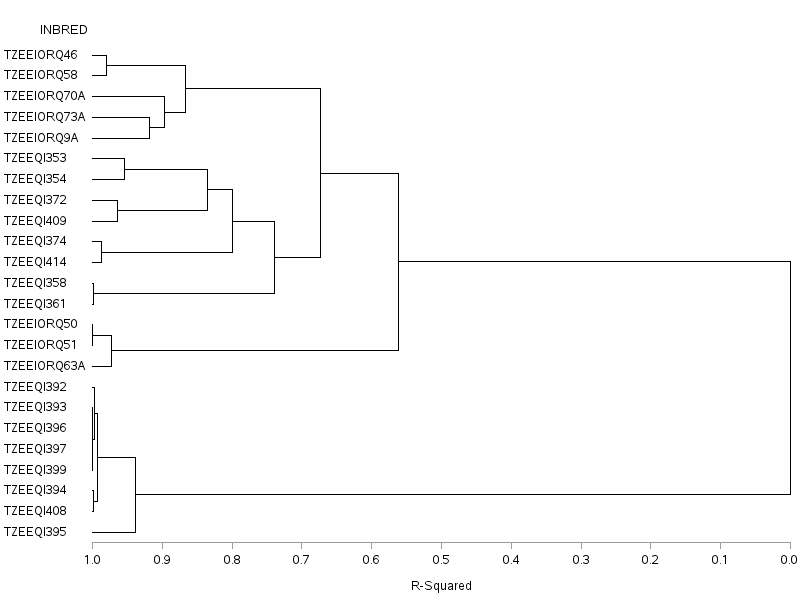


**Supplementary Figure 1**. Dendrogram showing the genetic relationship among the 24 inbred parents with SNP markers using Nei 1983 genetic distance methods and the Ward’s minimum variance clustering method.
